# Robust organ mapped dose: using multiple image registrations to identify deformation uncertainty in radiation dose mapping

**DOI:** 10.1088/1361-6560/ae4b01

**Published:** 2026-03-26

**Authors:** Christopher Thompson, Stina Svensson, Robin Prestwich, Christopher Pagett, John Lilley, Louise Murray, Ane Appelt, Michael Nix

**Affiliations:** 1 Department of Medical Physics, Leeds Cancer Centre, Leeds Teaching Hospitals NHS Trust, Leeds, United Kingdom; 2 RaySearch Laboratories, Stockholm, Sweden; 3 Department of Clinical Oncology, Leeds Cancer Centre, Leeds Teaching Hospitals NHS Trust, Leeds, United Kingdom; 4 Leeds Institute of Medical Research at St James’s, University of Leeds, Leeds, United Kingdom

**Keywords:** reirradiation, deformable image registration, robustness, dose mapping

## Abstract

*Objective*. To assist with reirradiation (reRT) treatment planning, we propose a robust organ-mapped dose (ROAD) method for cumulative dose estimation within critical organs-at-risks (OARs), incorporating deformable image registration (DIR) uncertainty via a dose resampling kernel derived from organ-specific independent DIRs. *Approach*. The discordance among three distinct DIRs, each of unknown accuracy, was used to estimate spatial uncertainty. For each voxel within an OAR, the discordance generated a per-voxel dose-resampling kernel. Two additional kernel expansions incorporated uncertainties not captured by inter-DIR discordance: the first ensured all returned dose originated within the OAR, while the second ensured all OAR dose voxels were sampled. The maximum dose within the kernel–OAR intersection was assigned to each voxel to yield a robust dose map. The approach was demonstrated for five pelvic, five head-and-neck, and five thoracic reRT cases using DIR-mapped background doses. Kernel generation was analysed by tracking kernel magnitude and its correlation with mean distance to agreement (MDA) and Hausdorff distance. Resulting dose distributions were compared with baseline mapped doses and a fixed-kernel robustness method. *Main results*. Analysis confirmed generally well-chosen DIRs but revealed residual errors beyond DIR discordance, detected by the additional kernel expansions. ROAD produced dose distributions comparable to fixed-kernel methods under low deformation uncertainty but demonstrated greater robustness in regions with large anatomical variation, particularly in the pelvis. ROAD reduced instances where mapped near-maximum doses underestimated original values, without increasing overall dose, by capturing uncertainty from organ filling and positional changes missed by fixed-kernel accumulation. *Significance*. Accurate cumulative dose estimation is critical for safe and effective reRT planning. The proposed ROAD framework explicitly incorporates voxel-level DIR uncertainty, providing a more reliable OAR dose estimate in regions with substantial anatomical change. This enhances confidence in reRT dose assessment and offers a practical, robust tool for clinical evaluation of cumulative organ doses.

## 1. Introduction

Accurate mapping of radiotherapy (RT) dose distributions between image-sets has broad applications, but our work specifically focuses on its critical role in planning and evaluation of reirradiation (reRT) as defined by Andratschke *et al* (2022). In the context of reRT, summed doses are used to ensure safe cumulative doses to organs at risks (OARs) while maintaining the efficacy of the second treatment course. Rigid image registration (RIR) is often used to map doses, however deformable image registration (DIR) can accommodate more marked anatomical changes (Ren *et al* 2019, Murr *et al* 2023) and has been shown to better represent the dose after transfer to a new image-set compared to RIR (Nix *et al* 2022, Thiong’o *et al* 2025). Anatomical and positional changes between treatment courses, however, can create significant uncertainty in dose accumulation (Brock *et al* 2017, Hardcastle *et al* 2024). Subtle deformation failures can also be hard to detect, rendering reliable dose mapping nontrivial (Wahlstedt *et al* 2023) with no ground truth for direct validation.

To safely implement DIR, a recent review by Nenoff *et al* (2023) recommended the development of tools to estimate and incorporate DIR uncertainties during dose accumulation. To date, clinical DIR adoption has relied on a combination of quality assurance approaches, including visual assessment, basic image similarity metrics, landmark tracking and structure comparison metrics on deformed regions of interests (ROIs) (Brock *et al* 2017, Ragul *et al* 2024). Validation of DIR is extremely challenging, with no consensus on the best approach. Common DIR performance metrics include mean distance to agreement, Hausdorff distance (HD), dice similarity coefficient (DSC) and target registration errors (Taha and Hanbury 2015, Muller *et al* 2022). These measures are incomplete and partly correlated, however use of multiple measures is still advised (Zhong *et al* 2024). Such metrics at best offer partial information on DIR quality and often do not localise uncertainties.

Tools to directly validate mapped dose or include a measure of dose mapping uncertainty have previously been investigated. Kainz *et al* (2021) introduced a dose volume histogram (DVH) overlay metric for DIR dose accumulation quality assurance. This technique estimates the uncertainty in DIR dose accumulation by comparing DVHs from multiple registrations. Their study quantified dose accumulation errors at the organ level for bladder and rectum in ten male pelvis cases. Takemura *et al* (2018) introduced a local uncertainty metric derived from candidate positions in uniform CT density ROI regions. Their metric was used to evaluate the reliability of dose accumulation by quantifying the uncertainty at each position within the deformation vector field.

Artificial intelligence (AI) based uncertainty quantification has also been proposed. Smolders *et al* (2023) presented deep learning methods to predict the localised uncertainty in DIR used in adaptive RT. The authors developed supervised and unsupervised neural networks to estimate the Gaussian uncertainty of deformable vector fields (DVFs). These methods were validated on lung cancer cases, showing high accuracy in predicting structure and dose uncertainties. Rivetti *et al* (2024) introduced a probabilistic multi-resolution image registration model using convolutional neural networks to estimate the uncertainty in DIR. Their model predicted a multivariate normally distributed dense displacement field and evaluated its performance against other DIR algorithms. The results demonstrated that the proposed model excels in both structure propagation and uncertainty estimation, achieving high accuracy and reliability. None of the above approaches have been incorporated into clinically available tools, and AI methods require clinical site-specific model development and validation. Moreover, they do not address the question of how to incorporate the estimated uncertainty when mapping dose deformably between image-sets.

The use of a fixed dimension dose resampling kernel to incorporate DIR uncertainty has previously been described (Mechalakos *et al* 2024, Thiong’o *et al* 2024, Thompson *et al* 2024) and is a simple but effective way to incorporate uncertainties. However, fixed-kernel dose-sampling methods suffer two limitations. Firstly, the fixed-kernel magnitude (KM) has to be chosen heuristically and may not be appropriate for all patients or treatment sites. Secondly, and most importantly, fixed-kernel methods do not account for local organ filling or positional changes, which can lead to significant localised failures in DIR.

Rather than attempting to validate any given DIR, or estimate the uncertainty of a single DIR directly, our ‘**R**obust **O**rgan m**A**pped **D**ose’ (ROAD) method relies on discordance between a number of different but plausible DIR methodologies to estimate local DIR uncertainty as demonstrated by others (Nenoff *et al* 2020). Thiong’o *et al* (2024) used DIRs with different starting conditions to define a set of plausible DIRs, which were used to produce a set of possible mapped doses and therefore a measure of dose uncertainty. Critically, with ROAD, we use a per-organ structure-only DIR (for each OAR) as one of the DIRs, focussing on discordance between a global DIR and OAR-specific DIR’s, to determine per-voxel dose resampling kernel dimension within each given OAR. For reRT we required ROAD to robustly map dose, specifically avoiding local underestimates but with minimal overestimates, relative to the original DVH. To this end we uniquely included two conditional kernel expansion processes, to capture additional uncertainty beyond the measured DIR discordance. The first applied when kernels fell entirely outside the OAR contour, with kernel expansions performed to ensure the returned ROAD dose originated in the OAR. The second kernel expansion ensured all doses associated with the OAR on the original scan had been sampled to at least one voxel in the reRT OAR. We demonstrate the validity of each part of the ROAD procedure and that ROAD overall operates as an independent uncertainty and hence robustness estimator, which does not rely on the inherent properties of the baseline DIR, validation metrics, or a given AI model. We present ROAD in the setting of reRT, with some example cases as a proof of principal.

## 2. Method

ROAD was performed independently for each OAR present on the original and reRT scans, using discordance between two global DIRs and one OAR-specific DIR to estimate per-voxel geometric uncertainty kernels, that were then used for robust dose mapping. While our uncertainty kernel estimation used discordance between three different DIR approaches, in principle, any number (minimum of 2) could be incorporated.

All DIRs used the RayStation Anaconda algorithm (Lorenzo *et al* 2024, Weistrand *et al* 2015). A 2.5 mm DIR grid was used to provide a fine resolution closely matched to the 3 mm dose grid. During dose mapping, each dose voxel was assigned a deformation vector interpolated from the DVFs onto the dose grid. We defined ‘original’ dose as existing on its original image-set (the historic treatment CT scan). A baseline DIR, using all structures as controlling OAR structures alongside image intensities, was used to map the original dose to a destination image-set (reRT treatment CT), creating a ‘baseline’ mapped dose.

### 2.1. Dose sampling kernel generation

**Figure 1.**
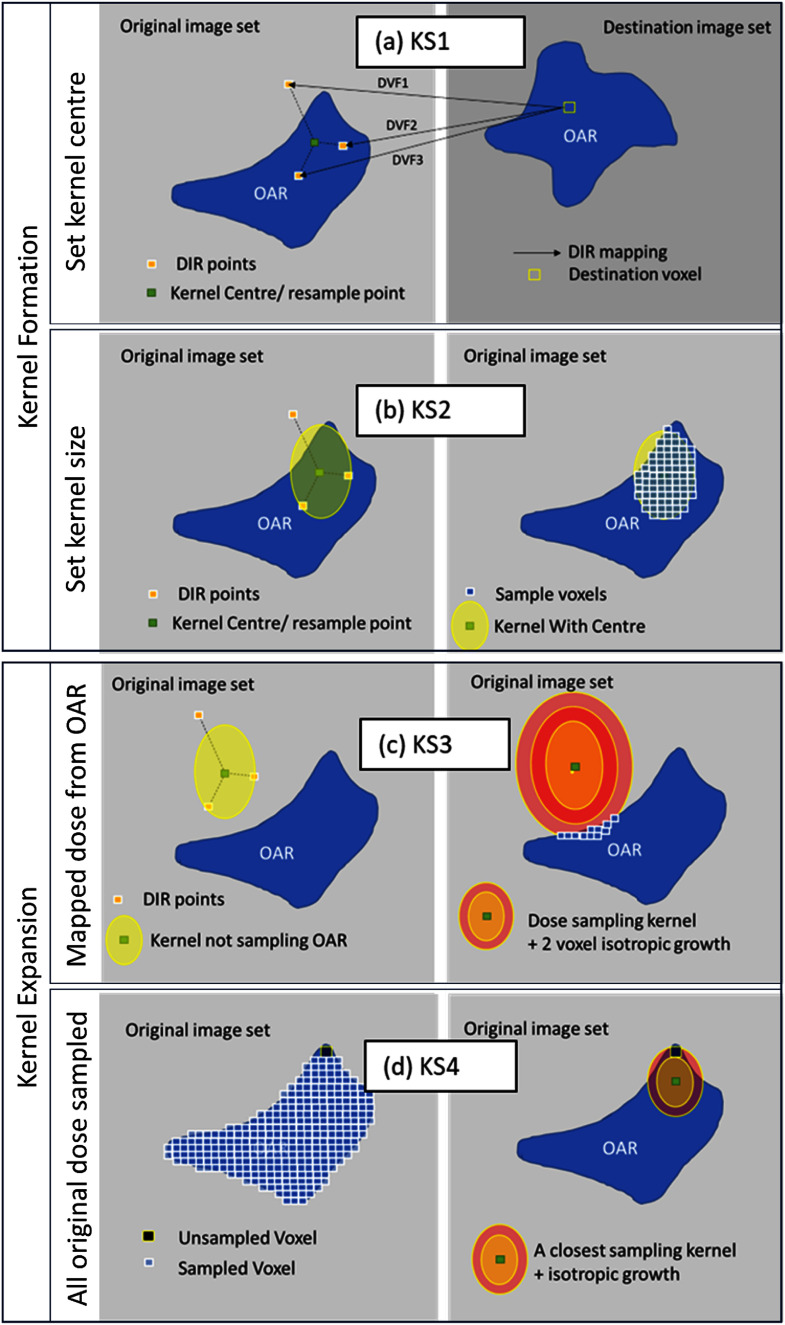
(a) Shows the KS1 process for determining a new kernel centre that is also used as a new primary dose resampling point. The three deformation vector fields (DVFs) define three unique points on the original image-set (DVFs being stored on a regular grid on the destination scan) with the outlier weighted down in the calculation of the kernel centre. (b) Shows KS2 kernel creation and deployment where kernel dimensions were computed from the weighted difference between the DVF points and kernel centre (left). Only dose voxels inside the intersection of the kernel and organ at risk (OAR) structure were returned as potential candidate doses for the destination voxel (right). (c) Shows KS3 the kernel expansion process adopted when a kernel did not sample any OAR dose voxels (left). Incremental isotropic kernel expansion continued until the kernel overlapped with the OAR structure (right) so that at least one dose voxel sample was taken from the original scan OAR contour. This ensured that sampled dose originated from the voxels associated with the OAR. (d) Shows KS4, the final kernel expansion process, which was applied after KS1-3 were completed for all destination image-set OAR dose voxels. During KS1-3 a record was kept of all the OAR original dose voxels sampled by the kernels. At KS4, sufficient unsampled OAR voxels triggered incremental isotropic expansions and resampling by kernels closest to the unsampled voxels. This incremental expansion was repeated until >99% of dose voxels were sampled.

An overview of the dose sampling kernel generation process can be found in figure 1. Alongside our baseline global DIR, that created a deformation vector field, DVF_baseline_, independent organ-specific DIRs were computed for each OAR. Binary structure masks for any part of a voxel inside the OAR assigned voxels to an OAR; fractional voxel contributions were not modelled. For these DIRs, which did not incorporate image intensity information, a single OAR was used as the controlling structure to generate the DVF_OAR_. By generating a DIR limited to a single OAR it was anticipated that differences would occur where the baseline DIR was influenced by conflicting OAR contours and/or image information, such as sliding organs. A third modified DIR was generated that did not use OAR contours but used only image intensity information (thus eliminating contour uncertainty from the DIR process), termed DVF_intensity_. While none of these DIRs represent ground truth, or have quantifiable uncertainty, their discordance should scale with uncertainty. By including two kernel expansion steps we ensure mapped OAR dose voxel originated from the original OAR and all the original OAR dose voxels map to the reRT image-set OAR for increased robustness. To explore the impact of each step in the proposed ROAD approach, a set of mapped doses were generated, with one for each of the 4 steps (KS 1–4) of the kernel generation and expansion process, as outlined below.

**KS1 (kernel centre):** DVFs were defined conventionally, as vectors pointing from a regular grid of origins on the (reRT) image-set, to a set of non-regular locations on the (original) image-set. Each voxel on the destination image-set (reRT scan) was the origin of mapping vectors pointing to different locations on the original scan. Therefore, for a given dose voxel in the destination image-set, all three DVFs were used to identify separate points (*p_i_*), with coordinates (*x_i_, y_i_, z_i_*), on the original image-set, from where dose could potentially have originated as shown in figure 1(a). The kernel centre was determined as the (weighted) mean of these original scan locations. The Euclidean distance from each of these points to their mean position, referred to as the distance to mean (DTM*_i_*), was computed. This DTM*_i_* value was then used to compute an inverse distance weighting factor (IDW*_i_*), to reduce the effect of an outlier DVF on both kernel centre position and kernel dimensions (KD) (as computed in **KS 2**),

\begin{equation*}{\mathrm{IDW}}{{\text{ }}_i} = \frac{1}{{1 + {\mathrm{DT}}{{\mathrm{M}}_i}}}{\text{ }}.\end{equation*}

A weighted kernel centre (kc) coordinate position was thus computed for the 3D dose kernel centre (where $l \in \left\{ {x,y,z} \right\}$):

\begin{equation*}{\mathrm{k}}{{\mathrm{c}}_l} = \sum\limits_i {\frac{{{\mathrm{ID}}{{\mathrm{W}}_i} \times {l_i}}}{{\sum {\mathrm{I}} {\mathrm{D}}{{\mathrm{W}}_i}}}} \end{equation*}

**KS2 (KD):** The per-voxel KD (where $l \in \left\{ {x,y,z} \right\}$), were computed as the weighted distance of the weighted standard deviations of the individual DVF sample points for *M* deformations (in our case *M* = 3).

\begin{equation*}{\mathrm{K}}{{\mathrm{D}}_l} = \sqrt {\frac{{\sum {\mathrm{IDW}}{{\text{ }}_i} \times {{\left({l_i} - k{c_l}\right)}^2}}}{{\frac{{M - 1}}{M}{\text{ }}\left( {\sum {\mathrm{ID}}{{\mathrm{W}}_i}} \right)}}} .\end{equation*}

This resulted in a 3D ellipsoid kernel per mapped dose voxel within each OAR. These kernels were modified by clipping them to their intersection with the original organ structure (i.e. dose voxels outside of the organ were not included in the dose resampling process), as shown in figure 1(b). Each voxel on the destination image-set was therefore associated with a kernel on the original dose distribution, derived from all three DVFs.

**KS3 (out of OAR expansion):** If kernels existed entirely outside the original OAR, all three DIRs had failed in a particular region, leaving no dose to sample to a particular reRT voxel under **KS 2**. To ensure that at least one voxel associated with the relevant OAR was sampled to each destination voxel, an incremental isotropic expansion of one dose voxel (3 mm in the current dataset) was applied to such kernels, until overlap with the original OAR structure was achieved, as shown in figure 1(c).

**KS4 (unmapped voxel expansion)**: To accommodate ‘true’ deformation beyond any applied DRR, KS4 expansions was triggered when more than 1% of the original OAR dose voxels remained unsampled after **KS 3.** In this case isotropic expansions were applied to the subset of kernels whose centres were closest to the unsampled voxels (figure 1(d)). The fraction of kernels expanded was set equal to the fraction of unsampled OAR voxels. Increasing the kernel radius increased the overlap between the kernels and the original scan OAR, thereby reducing the number of unsampled voxels. This expansion step was repeated until >99% of the original OAR voxels were sampled, or until 10 expansions in total had occurred (up to a +3 cm kernel radius).

### 2.2. Method evaluation—general aspects

For comparison with the ROAD method, a simple robust mapped dose using a single DIR and fixed isotropic kernel size (as described by Mechalakos *et al* ^16^) was generated. 3 mm fixed-kernels were centred on the point given by the baseline DIR or nearest point inside the OAR structure. We summarise the different approaches in table 1.

**Table 1.** Our proposed kernel steps for geometric and dosimetric analysis. In all steps, if the kernel generated dose was below the baseline deformation value, the baseline value was retained.

Kernel approach (KS)		
**Name**	**Description**	**Dose sampling**

Fixed 3 mm	A 3 mm spherical kernel, positioned at the point given by the baseline DIR or nearest point inside the OAR structure.	Max of samples taken from the intersection of the kernel and OAR. When kernel centre fell outside the OAR, kernel centre updated to closest point inside OAR.
KS1	Resampling at the kernel centres only.	Max of up to 2 samples: baseline mapped dose & kernel centre (if kernel centre inside OAR structures).
KS2	Resampling within kernel.	If kernel intersects with OAR, then max of samples, otherwise baseline mapped dose.
KS3	Kernel growth to ensure dose sample includes OAR voxels.	Max of samples with kernel expanded to ensure intersection with OAR.
KS4	Kernel growth to ensure >99% of original OAR dose voxels sampled.	Max of samples with kernel expanded to ensure >99% of original OAR sampled.

For ROAD methods after KS1, all previous kernel steps were included i.e. for KS4 dose accumulation KS1, KS2, KS3 and KS4 are all used. For methods KS1-3, it was possible for a kernel to fail to sample dose inside the OAR structure. If this occurred, or the kernel sampled maximum was lower than the baseline mapped dose, the mapped dose for that voxel took the baseline voxel dose value.

Robust doses were only computed inside OARs, considering each separately, with its organ-specific DIR. OAR ROAD doses were then combined with the baseline mapped dose outside the OARs, to produce the final robust dose distribution.

For each destination image-set OAR voxel, kernels defined a set of possible origin dose voxel values. The associated histogram represented a plausible distribution for the mapped dose at that voxel. We return the maximum sampled dose, to make a direct comparison to Mechalakos *et al* ^16^. This conservative approach is suitable for reRT, where organ maximum doses are the primary concern but alternative values (mean, median, percentile dose) may be more appropriate for other applications.

### 2.3. Method evaluation—geometric analysis

For each clinical site, average MDA and HD values were computed for structures generated by mapping original scan structures to the destination image-set using each DVF (e.g. DVF_baseline_ generated *S*_baseline_). Each mapped structure was then compared to the native structure on the destination image-set (*S*_destination_). For each structure, mean MDA and mean HD were computed from the three pairwise structure intercomparisons (*S*_OAR_ vs *S*_destination_, *S*_intensity_ vs *S*_destination_, and *S*_baseline_ vs *S*_destination_).

Correlation of KM to these metrics was evaluated after each of the kernel expansion steps, comparing mean-KM to the mean-MDA and maximum-KM to HD. A good correlation (>0.7) with MDA and HD at KS 2 would indicate KM values appropriate to the uncorrected deformation errors. Ideally this correlation would be maintained or improved during kernel expansions. Consequently, the impact of the kernel expansion steps (KS3 and KS4) on KM, relative to KS2, was evaluated. The degree of KM expansion beyond KS2 indicated the extent of organ deformation that had not been captured by the three selected DIR approaches, and hence the utility of the expansion process. KM was computed as the root mean square of *k*_d*x*_, *k*_d*y*_ and *k*_d*z*_ and evaluated (i) for OAR specific behaviour, (ii) for correlation with MDA and HD calculated for the mapped original OAR contours difference to the destination image-set native OAR contours, (iii) by visual inspection of KM overlaid on the destination image-set.

### 2.4. Method evaluation—dosimetric analysis

DVH statistics were extracted for all OARs, from the original, baseline mapped, fixed 3 mm and ROAD mapped dose distributions, as specified in table 1. ROAD mapped dose should always be equal to or higher than baseline mapped dose but may be lower than the original dose, if errors were not fully accounted for by the robust resampling.

We evaluated D_0.1cc_, D_5cc_ and mean dose for all OARs, and ROAD methods listed in table 1, determining:

(a) The number of mapped dose (*baseline* and *robust*) statistics more than 0.5 Gy below the values for the *original* dose distribution.
(b) A sum of all the doses below baseline.
(c) Overall median site dose increases above the baseline mapped dose.

Optimally, a robust dose approach should eliminate underestimated doses (a & b) while minimising the increase in mapped dose (c).

### 2.5. Computation times

Finally, we also evaluated the average time taken to generate the robust dose for an OAR in each of the three sites compared to the baseline dose mapping.

### 2.6. Patient cases

We evaluated ROAD on pelvis, lung, and head & neck reRT patient cases (five per anatomical site), treated with radical RT and subsequent reRT at Leeds Teaching Hospitals NHS Trust (LTHT). Time between treatments was 6 months to 3 years. For these cases, it is assumed that set up uncertainties were handled during plan delivery and are not explicitly dealt with by ROAD. Approval for retrospective use of patient data for research was granted by The Leeds Cancer Centre Computer Aided Theragnostics (LeedsCAT) research database (Research Ethics Committee reference 24/YH/0143). The details of the original treatment plans that were mapped to a reRT destination scan were:

• **Pelvic cases** had original prescription doses 46 Gy (*n* = 1) (prostate and nodes), 76 Gy (*n* = 1) and 37.5 Gy (*n* = 1) (prostates), and 52.6 Gy (*n* = 2) (prostate beds). OARs evaluated and used in DIR_baseline_ were rectum, colon, bladder, cauda equina and sacral plexus.
• **Lung cases** had original prescription doses: 55 Gy (*n* = 3) (lung SABR), 50 Gy (*n* = 1) and 55 Gy (*n* = 1) (lung). OARs evaluated and used in DIR_baseline_ were airways, lungs, brachial plexus, pericardium, oesophagus, spinal canal and trachea.
• **Head & neck cases** had original prescription doses 60 Gy (*n* = 1) and 70 Gy (*n* = 1) (nasal cavity), 60 Gy (*n* = 1) (sinus), and 70 Gy (*n* = 2) (tonsil). OARs evaluated and used in DIR_baseline_ were brainstem, larynx, mandible, optic chiasm, spinal canal, optic nerves, parotid glands and orbits.

To ensure optimal structure-based DIRs, manually generated structure geometries were reviewed by a single medical physics expert. Auto-contouring and DIR propagation were not used. The structure revisions checked clinical contours, for contouring accuracy and consistent approach between images. In particular, we ensured open-ended structure contours (e.g. spinal canal) stopped at an anatomically matched point, to avoid unreal structure-based deformations, driven by differences in contouring policy. All work was conducted in RayStation12B (research version).

## 3. Results

### 3.1. Geometric analysis

KMs for all patients are shown in table 2 for KS2 to 4 (KS1 being a kernel free step). Head and neck and thoracic organs showed similar KMs; albeit with a larger IQR in thorax, indicating greater geometric variability and hence registration uncertainty relative to head and neck. Pelvic organs, in particular bladder and colon, showed the largest KMs, and the most variability, with several outliers due to gross changes in bowel position and bladder filling between image-sets.

**Table 2.** Mean (IQR) kernel magnitude for the 5 head and neck, 5 thorax and 5 pelvis cases. KS2 with no kernel expansions; KS3 with kernel expansions to ensure dose is sampled from inside the organ structure; KS4 with additional kernel expansion to ensure 99% of organ voxels in the original organ structure are sampled. Also shown are the OAR mean correlation coefficients of MDA compared to the mean KM and HD compared to the maximum KM.

OAR	KS2 (mm)	KS3 (mm)	KS4 (mm)
Head and neck			

Brainstem	1.1 (3.0)	1.5 (3.0)	2.8 (5.2)
Larynx	2.1 (3.0)	2.4 (4.2)	3.6 (5.2)
Mandible	1.4 (3.0)	1.7 (3.0)	2.5 (5.2)
Optic chiasm	2.4 (4.2)	2.9 (4.2)	5.5 (4.8)
Optic nerves	2.5 (2.7)	3.2 (3.2)	4.7 (3.3)
Orbits	1.6 (3.0)	2.0 (3.0)	2.3 (4.2)
Parotid glands	1.4 (3.0)	1.6 (3.0)	2.9 (5.2)
Spinal canal	0.2 (0.0)	0.6 (0.0)	1.8 (5.2)
Mean	1.6 (2.7)	2.0 (3.0)	3.3 (4.8)
MDA correlation	0.88	0.89	0.72
HD correlation	0.30	0.10	0.37

Thorax			

Airways	1.7 (3.0)	2.2 (3.0)	3.0 (5.2)
Brachial plexus	5.8 (6.0)	6.3 (6.0)	8.5 (7.0)
Lungs	2.1 (3.0)	2.2 (3.6)	2.4 (4.2)
Oesophagus	2.6 (4.2)	2.9 (4.2)	3.2 (4.2)
Pericardium	3.2 (1.2)	3.3 (1.2)	4.0 (2.2)
Spinal canal	2.3 (3.0)	2.8 (3.0)	3.0 (3.0)
Trachea	2.0 (3.0)	2.2 (3.0)	2.8 (4.2)
Mean	2.8 (3.4)	3.1 (3.4)	3.8 (4.3)
MDA correlation	0.77	0.79	0.77
HD correlation	0.59	0.65	0.65

Pelvis			

Bladder	6.6 (6.0)	6.6 (6.0)	8.1 (7.0)
Cauda equina	0.8 (3.0)	1.3 (3.0)	2.7 (4.2)
Colon	10.2 (11.1)	11.1 (12.2)	12.7 (14.0)
Rectum	3.0 (1.2)	3.2 (1.2)	6.4 (3.7)
Sacral plexus	2.4 (4.2)	3.0 (4.2)	12.0 (9.4)
Mean	4.3 (4.8)	4.7 (5.0)	7.6 (7.1)
MDA correlation	0.91	0.91	0.66
HD correlation	0.70	0.73	0.91

KS3 kernels were larger than KS2 kernels for small organs, indicating destination voxels without ROAD doses prior to kernel expansion and that KS3 had a larger impact for these organs (e.g. chiasm). However, the impact of KS3 kernel expansion was generally small and less for larger OARs.

Across all sites, KS4 showed larger KMs (typically 3.0–10.0 mm) than KS2 and KS3, most notably in the pelvis, but with more modest increases in the head and neck and thorax. Violin plots of KMs are available in the supplementary material (figure S1.).

For head and neck, MDA correlated well with mean KM and with a modest drop when applying KS3 expansions. Correlation of maximum KM with HD was generally low with the exception of KS3, which showed a modest increase.

For thorax, MDA correlated reasonably with mean KM across all kernel expansions. Correlation of maximum KM with HD was improved compared to the head and neck slightly increasing at KS2.

For pelvis, MDA correlated very well with mean KM, but with a notable drop when applying KS4 expansions. Maximum KM, HD correlation conversely showed a significant increase at KS4.

**Figure 2.**
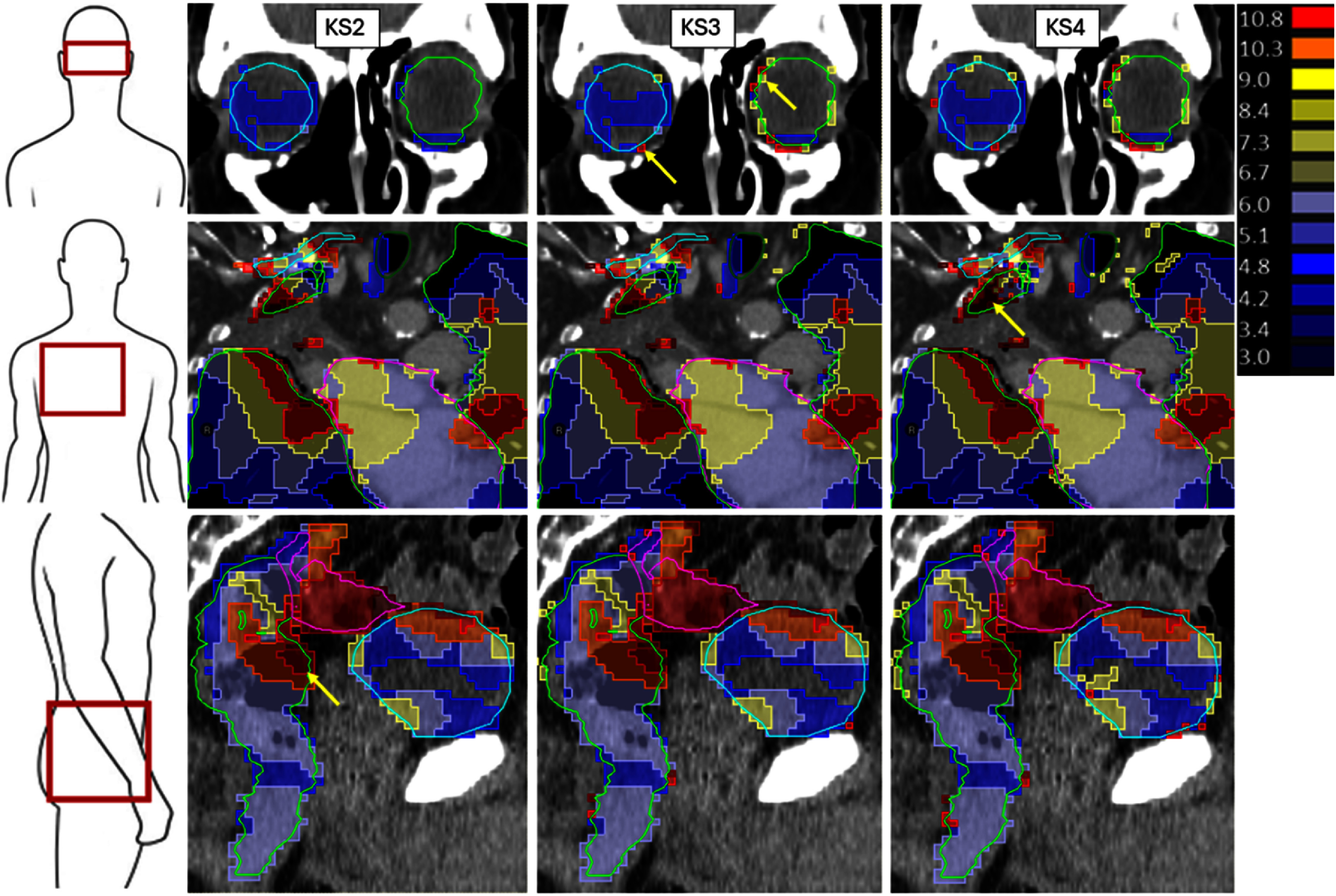
Heat maps of kernel magnitudes (KM) for different KS approaches with a scale in mm on the right. KS2 (left), KS3 (centre) and KS4 (right) KM (mm) overlaid on example patient destination images and native structure contours. Top row: left orbit (green), right orbit (blue), with arrows highlighting points where KM has increased between KS2 and KS3. Middle row: lungs (green), brachial plexus (blue), pericardium (purple) with an arrow indicating kernel expansions as KS4. Bottom row: rectum (green), colon (purple), bladder (blue), with an arrow to highlight where gas has locally increased rectum KM.

KM maps overlaid on CT images and structures (figure 2) in the orbits show the KM is predominantly either zero or 1 dose voxel (3 mm) with a slight concentration of non-zero kernels at the OAR edges. The KS3 expansions occur predominantly at the edges of the structure (yellow arrows) where partial voxel ownership will dictate whether a given voxel is assigned to an OAR.

For reference, Jacobian determinant data are also available in the supplementary material (table S1).

### 3.2. Dosimetric analysis

**Figure 3.**
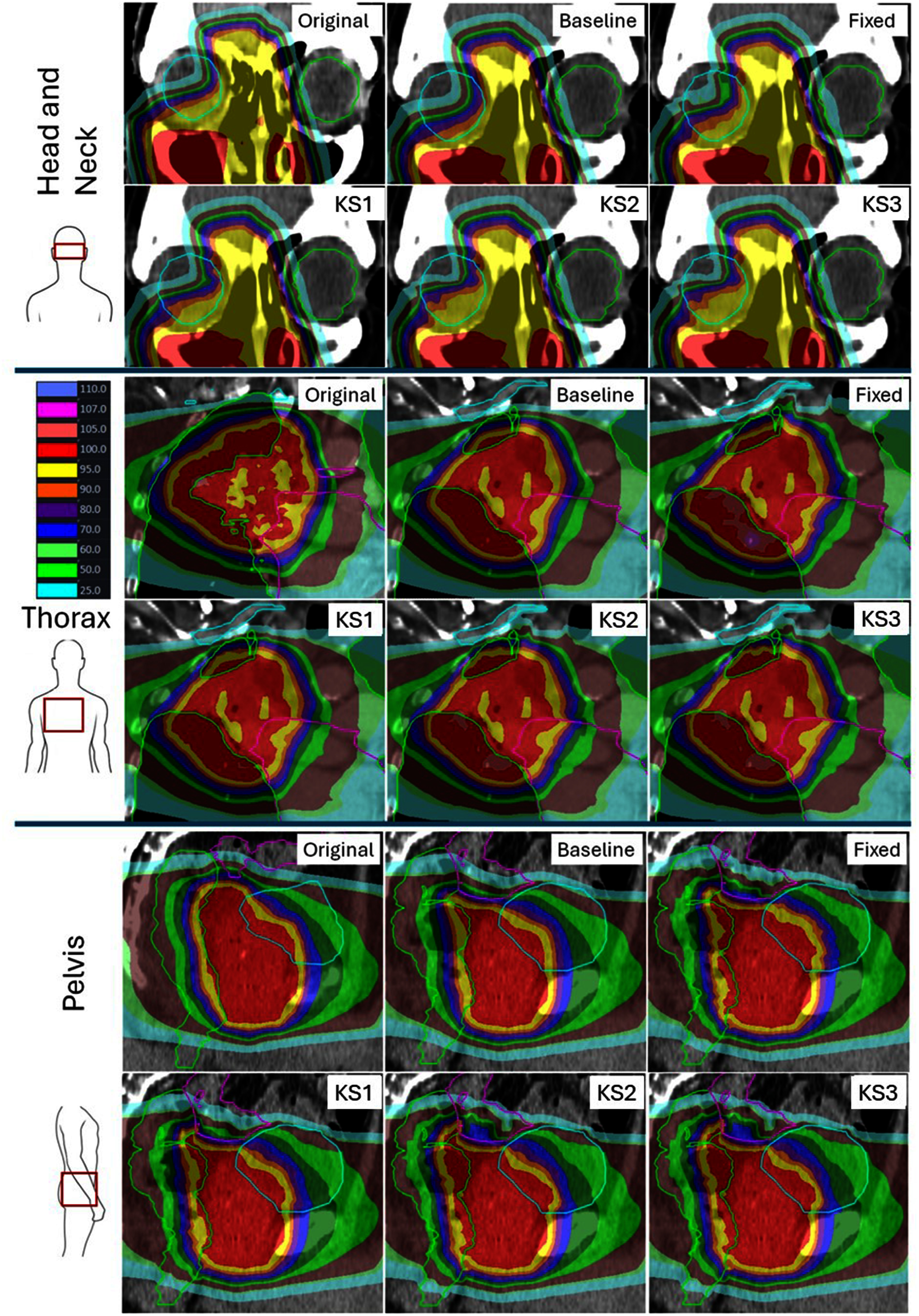
Example original dose, baseline mapped dose, fixed 3 mm kernel dose and robust mapping doses (KS1, 2&4), scale is shown as percentage of prescription dose. Doses were normalised to the prescription dose, which was 60, 55 and 37 Gy for the head and neck (top), thorax (middle, note the arrow to highlight the original image-set brachial plexus) and pelvis (bottom) plans respectively. Orientation as per figure 2.

**Figure 4.**
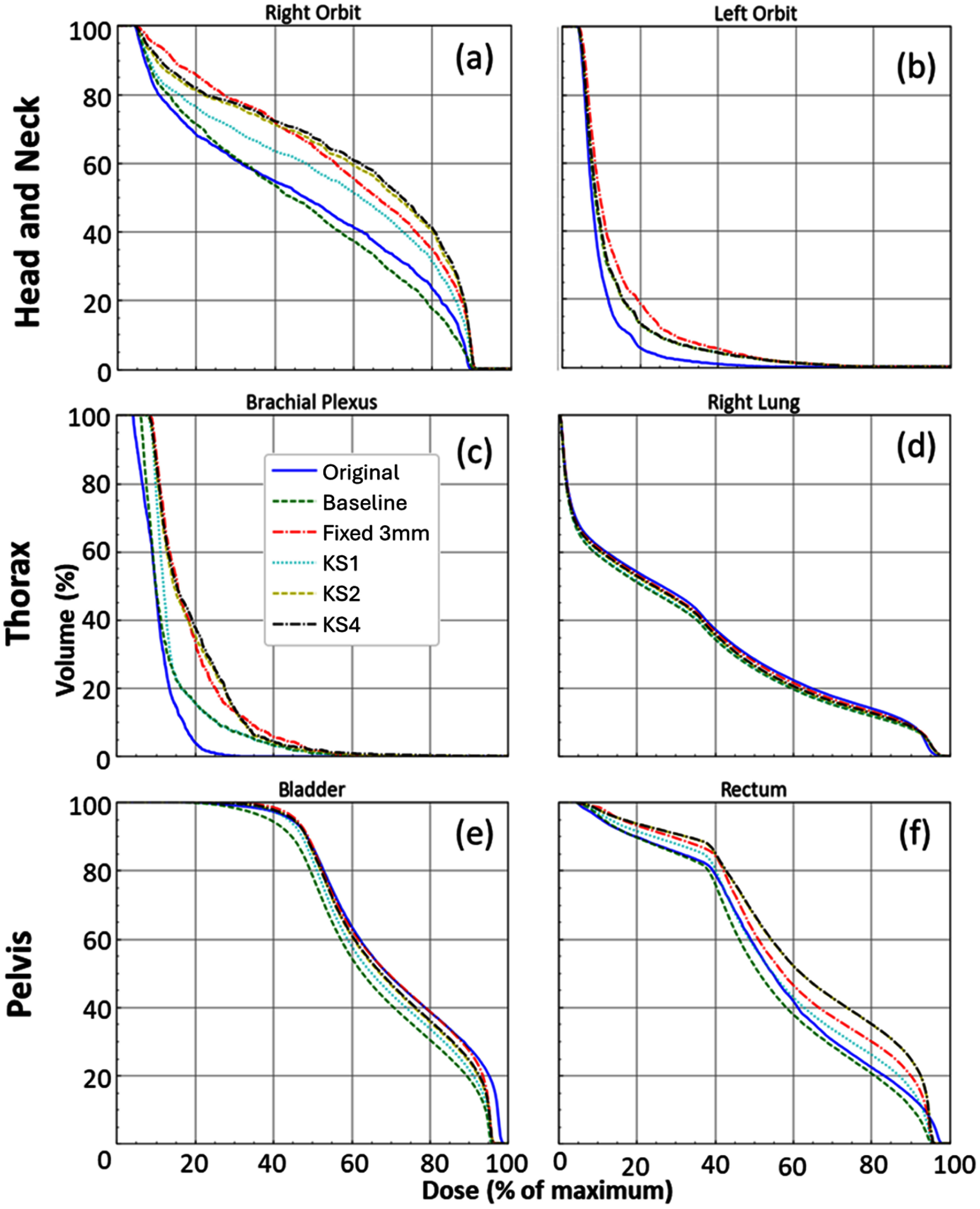
Selected example DVH data for the example patients included in figures 2 and 3. For head and neck (a) right orbit and (b) left orbit, for thorax (c) brachial plexus and (d) right lung, for pelvis (e) bladder and (f) rectum. A tendency towards more conservative dose estimates is generally evident across the progressive KS results, with qualitatively similar results in the other 4 cases.

KS1, simply returning the maximum of 2 doses taken from the baseline deformation location and the kernel centre, reduced the incidence of lower dose statistics in all sites, relative to baseline mapping. KS2, forming dose-kernels, further reduced underestimated dose statistics. KS3 made little impact on any of the reported dose metrics, indicating that most kernels overlapped with the OAR at KS2. KS4 achieved further reductions in underestimated dose metrics, indicating that there were original dose voxels which had not been mapped to the destination scan at KS3. With each additional KS step there was an associated increase in overall dose, and hence in mean dose statistics. Due to the limited impact of KS3 on doses, it is not included separately in the isodose overlays in figure 3 or DVH plots in figure 4, but is included in table 3.

**Table 3.** Impact of dose mapping approach on selected DVH metrics. Left three columns show the proportion (total dose/ count) of OARs where D0.1cc, D5cc and mean dose fell 0.5 Gy below the original plan dose statistic. The right three columns show median (over all patients and OARs) increase in dose compared to the baseline mapped dose. Results presented for the baseline dose, fixed 3 mm resampling kernel, KS1, KS2 (=KS1&2) and KS3 (=KS1,2&3) and KS4 (=KS1,2,3&4). Data is for 52 head and neck OARs (44 at 5cc due to OAR volume) with 8–11 per patient; 45 thorax OARs with 9 per patient; and 27 pelvic OARs with 5–6 per patient.

	Total mapped OAR dose below original value (Gy)/frequency (count)			Median increase in median dose above baseline (Gy)		
Metric	D_0.1cc_	D_5cc_	Mean dose	D_0.1cc_	D_5cc_	Mean Dose
Head and neck						

Baseline	−24.4/15	−17.5/9	−9.7/6	—	—	—
Fixed 3 mm	0.0/0	−0.6/1	−1.4/1	11.1	28.6	19.7
KS 1	−10.0/7	−7.3/5	−5.1/3	3.5	4.2	2.3
KS 2	−4.0/4	−5.1/2	−4.2/3	6.0	11.9	6.8
KS 3	−4.0/4	−5.1/2	−4.1/3	6.0	12.5	6.8
KS 4	0.0/0	0.0/0	−1.6/1	6.3	12.5	8.3

Thorax						

Baseline	−27.7/13	−9.3/6	−17.0/7	—	—	—
Fixed 3 mm	−1.6/1	0.0 /0	−7.4/2	12.0	5.7	14.6
KS 1	−5.8/3	−4.3/4	−13.6/6	2.8	0.6	4.2
KS 2	−1.1/1	−1.4/2	−9.3/4	6.5	1.3	10.4
KS 3	−1.1/1	−1.4/2	−9.1/4	6.5	1.3	10.4
KS 4	−0.5/1	−0.8/1	−8.1/3	13.4	2.5	10.4

Pelvis						

Baseline	−29.3/7	−49.2/10	−26.6/10	—	—	—
Fixed 3 mm	−5.6/1	−12.8/5	−15.0/5	4.2	31.3	17.6
KS 1	−9.1/2	−34.9/7	−20.5/9	3.9	12.0	2.9
KS 2	−2.5/1	−17.0/6	−15.8/6	4.2	21.6	2.9
KS 3	−2.5/1	−17.0/6	−15.7/6	4.2	21.6	2.9
KS 4	0.0/0	−2.1/1	−11.1/3	14.7	32.8	41.2

Fixed-kernel robust doses (table 3) show that the fixed-kernel approach also reduced the number of dose statistics lower than their original values, in the head and neck and thorax, and was comparable to KS4 in these sites. However, in the pelvis, where the highest increases in overall dose were also measured, KS4 outperformed the fixed-kernel approach.

### 3.3. Computation times

On our planning system, mean per patient times taken for the deformations and dose mapping including kernel resampling were ∼35 s for the baseline dose deformation ∼70 s for the fixed-kernel and ∼200 s for KS4. The longest times in kernel resampling were found when applied to larger OAR structures, due to the higher number of OAR voxels for evaluation.

## 4. Discussion

A major challenge facing any dose-mapping based on a DIR is the lack of a DIR ground truth, which renders the validation of DIRs, and deformation error estimation, non-trivial. However, quantification of DIR uncertainty has been recommended (Ren *et al* 2019) and explored (Takemura *et al* 2018, Kainz *et al* 2021, Rivetti *et al* 2024, Zhong *et al* 2024) alongside the application of DIR to dose mapping (Garcia-Alvarez *et al* 2025, Bender and Tome 2009, Hub *et al* 2012, Tilley *et al* 2013, Thiong’o *et al* 2024).

In the ROAD approach we do not attempt to directly measure the correctness or uncertainty associated with the DIR. We rather use the discordance of three DIR methods (including an OAR-specific structure-based DIR) to estimate the range of plausible DIR solutions at each voxel, to define per-voxel dose resampling kernels. Applying such kernels on a per-OAR basis, where dose accumulation errors are arguably most critical, permits dose-kernel expansion to (estimate and) account for the residual geometric uncertainty, beyond that encompassed by the DIR discordance.

The additional DIR methods included were selected specifically to address potential limitations of the baseline global hybrid-DIR, i.e. organ-specific DIR to eliminate image-driven or regularisation-driven errors and image-intensity only DIR to eliminate any contouring-driven errors.

We compared ROAD to a previous fixed-kernel approach (Mechalakos *et al* 2024, Thompson *et al* 2024), which shows fixed-kernels to be an effective way to incorporate uncertainties in mapped doses, particularly in head and neck and thorax RT. Returning the maximum kernel dose per-voxel, with fixed-kernel resampling, can however lead to a significant dose overestimates, which can be limiting when planning further treatment. Mechalakos *et al* (2024) measured a mean dose increase of 17% for the spinal canal, which is broadly in agreement with our results for a fixed 3 mm kernel (between 4.2%–31.3% across all OARs). A 3 mm kernel is justified based on the mean KS4 ROAD KMs in the head and neck and thorax (3.3 and 3.8 mm respectively). In the pelvis, the mean KS4 ROAD was 7.6 mm, suggesting a fixed 3 mm isotropic kernel applied to all OAR voxels was likely to be insufficient.

The relocation of the fixed-kernel centres outside the OAR contour to the nearest OAR associated voxel increased robustness but potentially could be over-conservative. Conversely, our ROAD KS3 expansions were terminated once a single OAR voxel was included in the kernel, representing a minimal expansion strategy. KS3 expansions had little impact on KM, indicating that the majority of KS2 ROAD kernels, based on 3 DIRs, overlapped their respective OAR contours. This rendered KS3 unnecessary for most voxels, as evidenced by the unchanged correlation with MDA and HD. However, the possibility of KS3 expansion provided reassurance that OAR doses were always sampled from the original OAR contour. Observed KS3 expansions were mainly limited to voxels at the edges of OAR structures (e.g. Figure 3—orbits), where contouring differences, proximity to the original contour boundary and differences in partial voxel ownership between scans would make kernels outside the OAR more probable.

KS4, accounting for unmapped original dose voxels, resulted in more kernel expansions, particularly in the pelvis. This indicated that some voxels’ ‘true’ deformation lay outside the kernel at KS3, implying that the DIRs did not fully capture the geometric uncertainty (as expected in challenging regions such as bowel). Hence KS4 served both as an additional robustness step, and an indicator of incomplete uncertainty estimation in the underlying DIR discordance. In this way, it was possible to use ROAD robustly, even in the presence of some unaccounted-for deformation error, and set a threshold for maximum acceptable KS4 expansion that indicated the underlying DIRs were insufficiently accurate for dose mapping.

There are certain scenarios in which kernel expansion must be handled carefully. In the case of missing tissue at reRT, kernel expansions could be an inappropriate approach (e.g. in the lung due to post surgery changes (figure 2)), although in this case perhaps expansion could be justified to account for the large resulting DIR uncertainties.

Generally, organs more subject to motion and volumetric changes, particularly within the pelvis, exhibited higher KS4 KM values and best captured local DIR uncertainty. However, it was possible for unreasonably large KS4 expansions to occur where individual loops of bowel had exchanged position. Hence, a limit of ten steps of KS4 expansion was used. The resultant KS4 KMs for the pelvis reflect the larger uncertainties, with a mean KM of 7.6 mm, approximately twice as large as both head and neck and thorax. The large KM values observed for colon were partly attributed to changes in bladder filling, which caused substantial positional shifts that baseline DIR could not accommodate.

Non-anatomical OAR boundaries (such as the spine-brainstem junction) or OARs characterised by low contrast (such as the brachial plexus) led to contouring variation between image-sets. This could lead to disagreement between the image-based and structure-based DIRs, resulting in artificially large KS2 kernels. Further non-DIR driven kernel expansion at KS3 and KS4, to accommodate incorrectly contoured voxels, then led to e.g. a 5 mm increase in KM for both the spine and brainstem at their interface, under KS4.

By considering the correlation of KM with MDA and HD (table 2), it was seen that while KS3 had minimal impact in most cases, KS4 tended to decrease the correlation between mean KM and MDA but increase the maximum KM and HD correlation. As MDA and HD were computed between deformed OAR contours and those defined directly on the destination scan, KS4 increased correlation of KM with HD, therefore evidencing appropriate kernel expansions at KS4. However, the decreased correlation with MDA indicated that kernel sizes on average were exceeding the residual error.

A ‘robust’ mapped dose should ideally exhibit DVH statistics at least as high as those for the original dose, but with a minimal overhead above that level. In the head and neck and thorax, near-maximum dose statistics (table 3), as typically used in the reRT setting, were robust by this definition for DIR mapped doses using either a fixed-kernel or KS4 (in total 0–1.6 Gy below original dose values). In contrast, the baseline DIR mapping was not robust (in total 9.3–27.7 Gy below). Each step of the ROAD process contributed to this robustness, primarily from KS1, KS2 and KS4 as discussed above. DVH plots (figure 4) show similar trends, with a notable dose increase at each KS step (with the exception of KS3). KS3 has also had minimal impact on mean dose increases (table 3) While KS3 is shown to be low impact, it does provide confidence that KS2 located kernel voxels within OAR contours. Despite a similar impact on underestimated doses, the fixed-kernel was associated with larger mean dose increases (5.7–28.6 Gy) compared to ROAD KS4 (2.5–13.4 Gy), and hence was robust but over-conservative.

In the pelvis, where deformations and uncertainty were larger, ROAD KS4 reduced the number and magnitude of underestimated point max statistics (in total 0–2.1 Gy below) compared to the fixed-kernel (in total 5.6–12.8 Gy below). The mean dose increases for point dose statistics were similar when comparing fixed-kernel and KS4, implying that a larger fixed-kernel would have further overestimated doses compared to KS4. Across all sites, none of the approaches were able to eliminate mapped mean organ dose statistics below their original values, despite all of them increasing mean reported doses. These persistently low mean doses could likely be attributed to OAR volume changes outside of the high dose region, due to filling or oedema, which could override the impact of robust dose mapping.

These dose results therefore indicated that in the absence of significant deformation challenges (as evidenced by the small KM in the head and neck and thorax), both our ROAD approach and a fixed-kernel with dimensions matched to the dose grid were effective in reducing under-reported point maximum doses, without producing over-conservative mapped doses. Therefore, for OARs which have minimal anatomical change and with well-defined anatomical boundaries, which aids both accurate contouring and drives deformation, adopting a fixed kernel approach was sufficient.

In the pelvis, however, ROAD represents a self-adaptive kernel-based dose mapping estimator, driven by the inherent uncertainty of independent per-organ structure-based DIRs. Furthermore, deformation errors unaccounted for by the underlying DIRs, and hence not included in KS3 kernels, were both estimated and accounted for by expanding kernels at KS4. Therefore, we considered that all KS steps (2, 3 and 4) were then necessary for a fully robust dose sampling in this scenario. While point maximum OAR doses were appropriately preserved, the use of a worst-case (maximum-value) resampling strategy resulted in increases in median dose. This occurs because each voxel is assigned the highest dose observed across possible samples, while the resulting maximum dose remains constrained by the true maximum present in the original dataset. In the reRT setting, OAR constraints are typically defined by point maximum dose criteria, making this approach acceptable. However, for applications where preservation of organ mean or median dose is critical, an alternative resampling strategy (rather than maximum-value sampling) would be required. The rectum in figure 3 is an excellent example of the need for local kernel estimation, as these regions were not robustly mapped by fixed-kernels or baseline-DIR mapping. ROAD KM maps also allowed visualisation of regions of unreliable DIR, which could enhance quality assurance, potentially allowing localisation of DIR failures, unlike DICE and other scalar metrics.

Robust mapped dose is particularly important in the case of reRT plan optimisation using a background dose (Nenoff *et al* 2023, Thompson *et al* 2023), where the local accuracy of the mapped dose is critical and will drive the reRT planning and dose distribution. Accurate and robust dose mapping here would allow safe and optimal reRT doses to be planned for patients receiving more than one course of RT, which is an increasingly common scenario. In the reRT setting, computation time may not be of huge concern, but to minimise the impact on workflow for time pressured work it may be appropriate to limit this approach to one or a few organs of dosimetric concern. Integration into the software may also enable more performant ROAD algorithms.

Other approaches to incorporate DIR uncertainties in dose mapping have been explored. For example, Alvarez. *et al* (2025) employed the inverse consistency error (Bender and Tomé 2009, Bender *et al* 2011) to generate a DIR error and applied that to a local dose gradient measurement for their robust dose mapping procedure. Thingo’o *et al* (2024) used 14 DIRs with different starting conditions to define a set of plausible DIRs and therefore provide a set of possible mapped doses to create a dose uncertainty measure. Wu *et al* (2010) addressed the contouring variability aspect via multiple human observers, generating a population of plausible organ contours for multiple DIR mappings. However, in clinical practice the additional contouring time would render this approach impractical, while also leaving inherent DIR error itself unaccounted for. All these methods do offer additional information regarding uncertainties, which are needed to make safe practical use of mapped doses. Fixed-kernel dose-sampling methods, as proposed for mapped dose robustness (Thompson *et al* 2023, Mechalakos *et al* 2024), are simple but suffer the limitations that the fixed-KM has to be chosen heuristically, is applied globally, and may not be appropriate for all patients or treatment sites. In contrast, ROAD could accommodate DIR errors (even beyond the discordance of the underlying DIRs themselves) and contouring errors and was able to robustly account for *localised* DIR failures which can be large in magnitude.

ROAD is a novel robust dose-mapping approach, through the combination of kernel-based dose resampling with DIR uncertainty estimation and kernel expansion methods. Whilst several of the individual elements have been explored previously, this has sometimes required non-clinical models or algorithms for uncertainty estimation, making translation challenging. We also extended the ROAD approach by using kernel expansions to ensure mapped dose was always sampled from within the original OAR structure, and that all dose associated with the original OAR structure was represented in the mapped OAR dose. We aimed to develop our method using existing clinically available DIR tools and scriptable functionality within a commercial TPS, by leveraging the independence of organ-specific structure-only DIR, as a comparator to global image intensity and hybrid DIR. It was designed such that complete DIR failure across all three DIR methods would manifest as large KS3 and particularly KS4 expansions. In such cases, ROAD would produce increasingly conservative dose estimates, signalling unreliable deformation rather than masking failure.

Our work was somewhat limited by the number of cases evaluated, with dosimetric evaluation therefore dependent on the actual dose distributions in sample of patient plans. However, ROAD is an analytical rather than data-driven method, so large samples were not required for the method development itself. We examined 105 organs (HN: 8 OARs × 5 patients, thorax: 7 OARs × 5 patients and pelvis 6 OARs × 5 patients) using representative clinical dose distributions. We also limited this investigation to a clinically relevant 3 mm dose grid, and 2.5 mm DIR grid for computational efficiency and clinical relevance. The DIR resolution should not impact the dose mapping results as the DIR resolution is finer than the dose grid; coarser DIR resolutions could add an additional interpolation error. Finer or interpolated dose grids may affect the local robust dose estimates, as could changes in DIR resolution both of which could form the basis of further work.

Our method, like any DIR-based mapping in the absence of ground truth deformation, can only be an estimator of deformation and associated uncertainty. We also acknowledge the statistical identification of outliers is not possible with only three DIRs, while the weighting applied improves the situation by reducing the impact of a DIR at odds with the other two, it does not stop overestimated kernels generated by a single DIR failure. Whilst we have leveraged independent DIRs to limit the potential for missed DIR error, the possibility remains, and careful review will always be required for clinical use. Furthermore, the dependence of ROAD on structures existing on the original and new images places demands on the consistency of contouring between image-sets. ROAD may overestimate KMs where the underlying anatomy has changed significantly, e.g. due to different bladder filling protocols, bowel contents, surgery or other volume-altering changes. In these cases, care is required, and a hard limit on permitted kernel expansions may be appropriate. In these scenarios, KS4, rather than a robust dose tool, could be used to flag cause for concern to the user. As we aim to demonstrate this as a tool within a single TPS, we are also restricting this to a single underlying DIR algorithm. Additional behaviours may be observed by altering the DIR algorithm itself, which could form the basis of further work. However, our inclusion/exclusion of controlling structures is designed to maximise DIR variance.

Our robustness approach only deals with those uncertainties generated by DIR variations within a single TPS; robustness to other sources of uncertainty such as delivery motion and independent DIR algorithms, are not included. Additionally, as ROAD only computes robust doses within OARs, the baseline DIR and mapped dose outside OARs would still require evaluation. However, these non-OAR tissues are rarely dose-drivers in RT planning, especially in the reRT setting. Given the promising fixed-kernel results within OARs with limited deformation, perhaps adopting a fixed 3 mm spherical kernel may be appropriate outside OAR structures. In this way, fixed kernels could be used in conjunction with ROAD KS1-3, reserving KS4 to individual OARs of particular concern, to add additional robustness or as an alert that DIR failure had occurred.

## 5. Conclusion

In cases with minor deformation uncertainty, a fixed-kernel of dimensions matching the dose grid was found to be sufficient to robustly map doses to a reRT scan. By using a DIR uncertainty-estimation kernel per OAR voxel, and a robust dose resampling strategy, ROAD was better able to accommodate larger deformation uncertainties which may be of greater clinical significance. Mapped doses were then found to be robust to DIR uncertainty whilst avoiding grossly over-conservative estimates. Using three independent deformations to underpin the approach allowed both DIR uncertainty and contouring differences to be incorporated into robust dose estimation. ROAD reduced the occurrence of DVH metrics below their original values, whist minimising overconservative dose mapping. Furthermore, the local nature of the ROAD approach allowed kernel size to increase where the underlying DIR uncertainty was higher, recovering robust dose locally, without unnecessarily increasing mapped dose globally. This more accurate representation of both the mapped dose distribution and its uncertainty will be critical to enabling optimisation of dose on a full background distribution from the original treatment in the reRT setting.

ROAD could be generalised to estimate kernels from more DIRs, if desired, or to incorporate uncertainty estimates from other sources (e.g. data-driven models). Dose resampling based on a kernel located at the mean of deformation vector origins added a level of robustness to the mapped dose estimates. Adding kernel-resampling and kernel expansions to ensure complete mapping of all voxels in both the source and destination image-sets further improved robustness.

Finally, the ability to visualise KM on the mapped dose provided further reassurance that ROAD had accommodated regions of uncertainty and provided clinical confidence in the use of mapped doses. The inclusion of ROAD mapping should permit a more informed and confident decision process in reRT scenarios involving deformable registration-based dose mapping for further treatment or evaluation, with value in clinical and research settings. The application of ROAD to different dose grid resolutions and other dose deformation situations encountered in RT will be the focus of future work.

## Data Availability

The data cannot be made publicly available upon publication because they contain sensitive personal information. The data that support the findings of this study are available upon reasonable request from the authors. Kernel Magnitude & Jacobian Data available at https://doi.org/10.1088/1361-6560/ae4b01/data1.
